# Effects of Bovine Lactoferrin on the Maintenance of Respiratory and Systemic Physical Conditions in Healthy Adults—A Randomized, Double-Blind, Placebo-Controlled Trial

**DOI:** 10.3390/nu15183959

**Published:** 2023-09-13

**Authors:** Hirotsugu Oda, Shutaro Kubo, Asuka Tada, Takumi Yago, Chihiro Sugita, Hiroki Yoshida, Tatsunori Toida, Miyuki Tanaka, Masahiko Kurokawa

**Affiliations:** 1 Innovative Research Institute, R&D Division, Morinaga Milk Industry Co., Ltd., 5-1-83, Higashihara, Zama 252-8583, Japan; 2 International BtoB Business Department, International Division, Morinaga Milk Industry Co., Ltd., 5-33-1, Shiba, Minato 108-8384, Japan; 3 Department of Biochemistry, Graduate School of Clinical Pharmacy, Kyushu University of Health and Welfare, 1714-1, Yoshino, Nobeoka 882-8508, Japan; 4 School of Pharmaceutical Sciences, Kyushu University of Health and Welfare, 1714-1, Yoshino, Nobeoka 882-8508, Japan

**Keywords:** lactoferrin, physical conditions, respiratory symptoms, systemic symptoms, plasmacytoid dendritic cells

## Abstract

Objectives: We investigated the effects of bovine lactoferrin (LF) on the maintenance of the respiratory and systemic physical conditions. Methods: A randomized, double-blind, placebo-controlled trial was conducted. Healthy adults at Kyushu University of Health and Welfare ingested a placebo or bovine LF (200 mg/day) for 12 weeks. The primary endpoints were the total respiratory and systemic symptom scores. The secondary endpoint was the activity of plasmacytoid dendritic cells (pDCs) in peripheral blood. Results: A total of 157 subjects were randomized (placebo, *n* = 79; LF, *n* = 78), of whom, 12 dropped out. The remaining 145 participants were included in the full analysis set (placebo group, *n* = 77; LF group, *n* = 68). The total scores for respiratory and systemic symptoms during the intervention were significantly lower in the LF group than in the placebo group. The expression of CD86 and HLA-DR on pDCs was significantly higher in the LF group than in the placebo group at week 12. Adverse events were comparable between the groups, and no adverse drug reactions were observed. Conclusions: These results suggest that orally ingested LF supports the normal immune system via maintaining pDC activity, and maintains respiratory and systemic physical conditions in healthy adults.

## 1. Introduction

Dendritic cells (DCs) integrate the innate and adaptive immunity. Plasmacytoid dendritic cells (pDCs) express Toll-like receptor (TLR)-7 and TLR9, detect viral single-stranded RNA (ssRNA) or CpG DNA, and produce Type I interferons (IFNs). pDCs upregulate the expression of costimulatory molecules such as CD86, and major histocompatibility complex type II (MHC II) molecules such as human leukocyte antigen (HLA)-DR upon their activation. Type I IFNs stimulate downstream broad and diverse IFN-stimulated genes subsequently, and induce an antiviral state in the host cells. Type I IFNs also activate myeloid DCs, natural killer (NK) cells, CD4+ helper T cells and CD8+ killer T cells, and B cells [1,2]. Therefore, pDCs function as the leaders in antiviral immunity, and modulate innate and adaptive immunity. pDC depletion worsens manifestations caused by respiratory syncytial virus infections in vivo [3]. In humans, IFN-α plays an important role in suppressing influenza virus infection [4,5]. These findings suggest that keeping pDC activity in a suitable state is preferable for alleviating respiratory and systemic symptoms. pDCs are detected not only in the peripheral blood, but also in the mucosa-associated lymphoid tissues in the upper and lower digestive tract, such as the tonsils, Peyer’s patches, and mesenteric lymph nodes [6,7,8]. Therefore, ingested food components may modulate pDC activity.

Lactoferrin (LF) is a cationic iron-binding glycoprotein present in the exocrine secretions including milk, saliva, and tears, and plays an important role in the first line of host defense. Currently, LF isolated from cheese whey or skim milk produced from bovine milk is widely used as a functional ingredient in food products such as infant formula, yogurt, beverage, and supplements [9]. LF upregulates the intracellular expression levels of IFN-α in pDCs, and cell surface expression levels of CD86 and HLA-DR on pDCs in the peripheral blood mononuclear cells (PBMCs) in the presence of ssRNA in vitro [10]. LF intake enhances the intracellular IFN-α expression levels in pDCs and cell surface CD86 expression levels on pDCs in the PBMCs from healthy adults [11]. Oral administration of LF enhances IFN-α/β production in the Peyer’s patches and activates spleen NK cells [12], and augments CD4+ helper T cells, CD8+ killer T cells, and IgA+ plasma cells in the Peyer’s patches and lamina propria in vivo [13]. The intake of LF induces the gene expression of type I IFNs in the large intestine, and activates NK cells, CD4+ helper T cells, and CD8+ killer T cells in the peripheral blood in humans [14,15,16]. In addition, oral administration of LF alleviates lung and intestinal inflammation caused by influenza virus infection in vivo [17,18]. In healthy individuals, LF intake suppresses respiratory and systemic symptoms [19,20]. These findings suggest that LF intake maintains pDC activity in a suitable state, and modulates the innate and adaptive immunity, leading to the alleviation of respiratory and systemic symptoms. In the present study, we investigated the simultaneous effects of LF ingestion on respiratory and systemic symptoms and the activity of pDCs in the peripheral blood using a randomized, double-blind, placebo-controlled trial.

## 2. Materials and Methods

### 2.1. Trial Design and Ethical Approval

This was a randomized (1:1), double-blind, placebo-controlled trial conducted between August and November 2022 at the Kyushu University of Health and Welfare in Miyazaki, Japan. The trial was performed in accordance with the current revision of the Declaration of Helsinki [21] and the Ethical Guidelines for Medical and Health Research Involving Human Subjects [22]. The protocol and informed consent forms were reviewed by the Institutional Review Board (IRB) of Kyushu University of Health and Welfare and approved on 10 June (approval number: 22-008). This trial was registered in the University Hospital Medical Information Network (UMIN) Clinical Trials Registry on 17 June 2022 (registration number: UMIN000048087). This protocol is available in Japanese from the corresponding author upon reasonable request.

### 2.2. Subjects

The eligible subjects were healthy adults between 18 and 65 years of age from Kyushu University of Health and Welfare. Subjects who met any of the following criteria were excluded from participation: subjects who regularly consume foods containing LF, subjects who plan or wish to become vaccinated or donate blood during the test period, extremely heavy drinkers (more than 60 g/day of alcohol), subjects who cannot sleep at night owing to irregular work or other reasons, subjects who take medicines or functional foods during the pretest period, which might affect their respiratory and systemic symptoms and/or immune function, subjects who cannot stop taking these medicines or functional foods during the test period, subjects who have serious disease in the liver, kidney, heart, lung, gastrointestinal tract, blood, endocrine system, or metabolic system, etc., or those who have a serious medical history of these, subjects having chronic disorders, subjects who are positive in the blood examination for Treponema pallidum, hepatitis B virus (HBV) antigen, hepatitis C virus (HCV) antibodies, or human immunodeficiency virus (HIV), subjects who need the application of medicine containing steroids, subjects having a milk allergy, subjects who have a medical history of drug allergy or severe food allergy, subjects who are pregnant or under lactation, or who are expected to be pregnant during the study, subjects who donated 400 mL of their blood within 12 weeks before the beginning of test-food intake, or not less than 200 mL of their blood during the pretest period, or others who have been determined as ineligible for the research subject of this study, judging from the principal researcher/doctor’s opinions on the findings of their background, physical observations, medical checkup, physical/clinical examination, and so on. For the sample size, based on an estimation of type I error (α) of 0.05, type II error (β) of 0.8, difference (δ) of 50, standard deviation (σ) of 100, and sample size ratio between groups of 1, we evaluated the sample size as 134 (67 in each group) using G*Power (version 3.1.9.7) and set the target sample size as 160 (80 in each group), considering the possible dropouts.

### 2.3. Intervention

After IRB approval, the principal researcher/doctor fully explained the details of the trial to the subjects in accordance with the informed consent form, and written informed consent was obtained from all subjects. The principal researcher/doctor enrolled 160 healthy subjects based on a background questionnaire and negative blood examination results for Treponema pallidum, HBV, HCV, and HIV. The subjects were randomly allocated to the placebo or LF group. They licked two tablets (placebo or 200 mg of LF per day) for a few tens of seconds and swallowed them afterward during the 12-week intervention. During the intervention, the subjects recorded their tablet intake, respiratory symptoms (throat discomfort, hoarseness, chest congestion (sputum), sneezing, runny nose, and plugged nose), and systemic symptoms (feeling feverish and malaise) in a diary. These symptoms were scored on an 8-point Likert scale from 0 (do not have this symptom) to 1 (very mild), 3 (mild), 5 (moderate), and 7 (severe), referring to the Wisconsin Upper Respiratory Symptom Survey and Jackson scale [23]. The principal researcher/doctor contacted the subjects during and after intervention to verify the validity of the record. Blood samples were collected before and after the 12-week intervention.

### 2.4. Endpoints

The primary endpoints were total scores for respiratory and systemic symptoms. The total scores of respiratory and systemic symptoms were calculated by summing the scores of individual symptoms obtained during the 12-week intervention. The secondary endpoints were expression of HLA-DR and CD86 on pDCs. Exploratory scores for respiratory and systemic symptoms in each 4 weeks were evaluated. Cumulative number of days of respiratory and systemic symptoms were also extracted from the adverse events record. The principal researcher/doctor discussed and evaluated all items.

### 2.5. Safety Assessment

Any unfavorable and unintended signs, symptoms, or diseases observed during the intervention were defined as adverse events. Adverse events were evaluated using the Revised National Cancer Institute Common Toxicity Criteria Version 4.0, and events in which a causal relationship with the intake of the test foods could not be ruled out were designated as adverse drug reactions.

### 2.6. Randomization, Allocation, and Blinding

The test food in the LF group was a round, red-orange tablet (weight 250 mg, diameter 9.1 mm, and thickness 4.8 mm) identical to the commercial product ‘Lactoferrin Original’ (Morinaga Milk Industry, Tokyo, Japan). This tablet contained 100 mg of bovine LF, maltitol, indigestible dextrin, dextrin, and hydrogenated rapeseed oil. Iron content in the LF was quantified by inductively coupled plasma optical emission spectrometer (18 µg/100 mg, corresponding to approximately 12.9% iron saturation). In the placebo tablet, LF was replaced with dextrin, and the tablet color was adjusted with food coloring. The tablets were similar in appearance, smell, taste, and packaging. An independent allocation manager confirmed that they were indistinguishable at the time of allocation and code breaking. The allocation manager produced a computer-generated allocation table with a block size of four (1:1 ratio), and numbered the test foods consecutively in accordance with the tables. The allocation tables were sealed in an opaque envelope and kept sealed until code breaking. The investigators numbered the enrolled subjects and assigned them to each test food item with a corresponding number. The investigators and participants were blinded until code breaking. Code breaking was performed after locking the database and the statistical analysis plan.

### 2.7. Blood Sampling

A Vacutainer Cell Preparation Tube (BD, Franklin Lakes, NJ, USA) was used to collect peripheral blood, and peripheral blood mononuclear cells (PBMCs) were isolated according to the manufacturer’s instructions. Isolated PBMCs were immediately used to analyze CD86 and HLA-DR expression on pDCs.

### 2.8. Measurements of Expression of CD86 and HLA-DR on pDCs

Referencing a previous in vitro study [10], PBMCs were stained with a fluorescent dye and fluorescent conjugated antibodies: BD Horizon Fixable Viability Stain 780 (FVS780) viability dye, PE-Cy7 Mouse Anti-Human CD123 (BD, 560826), BB515 Mouse Anti-Human Neuropilin-1 (CD304) (BD, 566036), and APC Mouse Anti-Human CD86 (BD, 555660) following the manufacturer’s instructions, and then fixed with 4% paraformaldehyde. PBMCs were cultured at 1 × 10^6^ cells/mL in RPMI-1640 (Sigma-Aldrich, St. Louis, MO, USA) supplemented with 5% human AB serum (Sigma-Aldrich), 2 mM Glutamax (Thermo Fisher Scientific, Waltham, MA, USA), and 1% penicillin/streptomycin (Thermo Fisher Scientific), and stimulated in the presence of 10 µg/mL TLR-7/8 agonist R848 (InvivoGen, San Diego, CA, USA) for 4 h. Afterward, the cells were harvested and stained with FVS780, PE-Cy7 Mouse Anti-Human CD123, BB515 Mouse Anti-Human Neuropilin-1 (CD304), and PE Mouse Anti-Human HLA-DR (BD, 556644) as described above. Data were collected using CytoFLEX (Beckman Coulter, Brea, CA, USA) and analyzed using the FlowJo version 10 software (BD). Isotype controls (BD) were used to check the background caused by nonspecific antibody binding. pDCs were defined as CD123+CD304+ subsets, and CD86 and HLA-DR were used as activation markers for pDCs.

### 2.9. Statistical Analysis

Analyses of the efficacy were performed on the full analysis set (FAS). Consent withdrawal, eligibility violation at the start of intervention, and lost to follow-up were excluded. The total scores for respiratory and systemic symptoms were evaluated using the Mann–Whitney U test. To avoid the multiplicity issue, significant difference in both scores was required for the total significant difference. The geometric mean fluorescence intensity (MFI) of CD86 and HLA-DR on pDCs was logarithmically transformed to obtain normally distributed data, and the values at week 12 were analyzed using analysis of covariance (ANCOVA), with adjustments for the values at week 0. The values at week 0 and week 12 were also analyzed using the paired *t*-test. EZR software Version 1.40 (Saitama Medical Center, Jichi Medical University, Saitama, Japan) was used for the statistical analysis [24], and a *p* value < 0.05 was considered statistically significant.

## 3. Results

Participants were recruited from 20 June to 24 June 2022, and the 160 subjects provided informed consent. Among them, three withdrew consent. A total of 157 subjects were randomized and allocated to two groups (placebo, *n* = 79; LF, *n* = 78). The baseline characteristics of the groups were comparable (Table 1).

**Figure 1:**
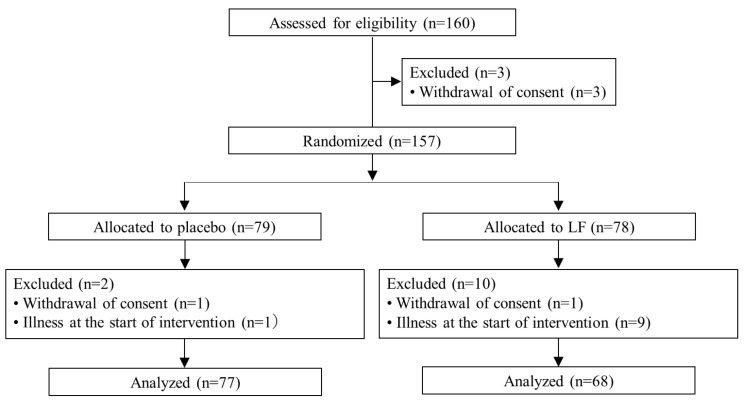
Consort flow diagram of the study.

After randomization, two subjects withdrew their consent, and ten subjects dropped out due to the onset of illness at the start of the intervention (chronic diarrhea, *n* = 1; chronic rhinitis, *n* = 4; chronic fatigue, *n* = 1; COVID-19, *n* = 4). The resulting full dataset was used for analysis (placebo, *n* = 77; LF, *n* = 68) (Figure 1).

Subjects started ingesting test foods on 8–10, 12, or 15–18 August 2022, and finished ingesting on 30–31 October, 1, 3, or 6–9 November 2022 (12 weeks). The median (interquartile range) intake rates of the test foods were 92.9% (83.3–96.4%) in the placebo group and 95.2% (86.9–100.0%) in the LF group, with no significant differences.

With regard to the primary endpoints, the total scores for respiratory and systemic symptoms in 12 weeks were significantly lower in the LF group than in the placebo group (Table 2 and Table 3). To speculate the required period to exert the effects, the scores in each 4 weeks were also evaluated exploratorily. The scores in weeks 1–4 did not show significant difference, but those in weeks 5–8 and weeks 9–12 were significantly lower in the LF group than in the placebo group.

Regarding secondary endpoints, the expression of CD86 and HLA-DR on pDCs was significantly higher in the LF group than in the placebo group at week 12 (Table 4). In both groups, the expression of CD86 and HLA-DR was lower at week 12 than at week 0.

During the intervention, a total of 115 subjects experienced adverse events, including the above symptoms, menstrual pain, abdominal discomfort due to excessive eating or drinking, muscle pain due to exercise, and not feeling well owing to a lack of sleep (placebo, 54/78 [69.2%]; LF, 61/77 [79.2%]), and no significant differences were observed between the groups. No adverse drug reactions were observed. Cumulative number of days of feeling feverish, malaise, chill, cough, hoarseness, plugged nose, and head congestion during the intervention were fewer in the LF group than in the placebo group (Table S1).

## 4. Discussion

We investigated whether the oral ingestion of LF (200 mg/day) affected respiratory and systemic symptoms, and the activity of pDCs in the peripheral blood in healthy adults. The total scores for respiratory symptoms, such as throat discomfort, hoarseness, chest congestion (sputum), sneezing, runny nose, or plugged nose, and systemic symptoms, such as feeling feverish or malaise, were significantly lower in the LF group than in the placebo group. A similar tendency was observed in the adverse events record. Alleviative effects of LF were clearer in weeks 5–8 and weeks 9–12 than in weeks 1–4 against both respiratory and systemic symptoms. Therefore, ingestion of LF for more than 4 weeks may be preferable to enjoy the benefits. Previous trials have also demonstrated that LF suppresses respiratory and systemic symptoms in healthy adults and children, which supports the results of the present trial [19,20]. As hundreds of exogenous antigens cause respiratory and systemic symptoms, it is difficult to precisely identify the causes of these symptoms. The Wisconsin Upper Respiratory Symptom Survey and Jackson scale are scientifically validated questionnaires [23]. Each country’s version has been published, including the Japanese version, which is widely used to assess respiratory and systemic symptoms worldwide. To reduce the burden on subjects, some of the listed respiratory and systemic symptoms were converted to plain terms, and investigated in the current trial.

The expression of CD86 and HLA-DR on pDCs was lower at week 12 than at week 0 in both groups, and was significantly higher in the LF group than in the placebo group at week 12. TLR7 function in PBMCs is lower in the dark season (October–March) than in the sunny season (April–September) [25]. Therefore, TLR7 function in pDCs may be lower at week 12 (fall) than at week 0 (summer), and LF intake may enhance TLR7 function in pDCs as previously reported [26], and affect pDC activity. Our previous study also reported that LF intake (200 mg/day for 4 weeks) enhanced IFN-α and CD86 expression on pDCs [11]. Considering the current and previous observations that suggested activation of NK cells, CD4+ helper T cells, CD8+ killer T cells, and B cells by LF [12,13,14,15,16], it is considered that ingested LF modulates the entire immune system through maintaining pDC activity, and contributes to the maintenance of respiratory and systemic physical conditions.

Endogenous LF is always present in exocrine secretions such as tears and saliva. Therefore, it is unlikely that LF itself extraordinarily stimulates the immune system. In fact, LF upregulated only CD86 expression on pDCs when there was no ssRNA present, but upregulated IFN-α, HLA-DR, and CD86 expression on pDCs in the presence of ssRNA in vitro [10]. This suggests that LF activates pDCs by enhancing the sensing of pDCs to exogenous antigens that contain TLR ligands when these antigens exist in the environment, contributing to the rapid clearance of these antigens. As the chronic production of type I IFNs by pDCs is a risk factor for autoimmune diseases [27], it is reasonable from the perspective of safety that LF activates pDCs when necessary. In addition, ingestion of LF reduced the production of inflammatory cytokines (IL-6, TNF-α) from peripheral blood cells in healthy adults [28]. Therefore, it is unlikely that ingestion of LF causes a cytokine storm.

Ingestion of exogenous LF may compensate for the effects of endogenous LF. Orally administered LF acts on the upper and lower digestive tracts, and also on the blood cells via the sublingual mucosa [29]. As pDCs are detected in the peripheral blood, tonsils, and small intestine, subjects in the current trial licked the test tablets for a few tens of seconds and swallowed them afterward, expecting to affect pDCs in the peripheral blood, tonsils, and small intestine. We used the same tablets in a previous trial to confirm the activation of pDCs [11]. Therefore, orally ingested LF may activate pDCs in the peripheral blood, tonsils, and small intestine. Considering in vivo kinetics, these effects are expected to be extended to chewable or liquid LF products. Contrarily, it may be difficult to expect the same effects in LF products whose in vivo kinetics are modified so as not to remain in the upper digestive tract. At least, there is currently no clinical evidence for pDC activation or reduced respiratory and systemic symptoms with enteric-coated products.

In this trial, the prevalence of adverse events was comparable between groups, and no adverse drug reactions were observed. Therefore, LF was considered safe, as shown in previous studies [11,19,20]. Furthermore, LF has a long history of ingestion as a component of milk and dairy products, and is classified as generally recognized as safe (GRAS) in the USA and as a Novel Food in the EU; thus, its safety is reliable.

This trial was conducted during late summer and fall in healthy men and women aged 18–38 years. In previous trials, TLR7-mediated responses in pDCs were enhanced by LF intake in elderly women [26], and suppressed respiratory and systemic symptoms in healthy subjects aged 20–52 years [20]. Therefore, LF intake may maintain pDC activity, alleviate respiratory and systemic symptoms, and contribute to the maintenance of physical conditions, regardless of sex and age.

The limitations of the present trial were bias in age distribution, season, lack of objective evaluation to identify the cause of respiratory and systemic symptoms, and lack of examination of proinflammatory or anti-inflammatory cytokine production. Investigations involving subjects with diverse backgrounds, in other seasons, or using objective measurements of respiratory and systemic symptoms and cytokine production are required to further improve the reliability of the results.

## Figures and Tables

**Table 1:** Baseline demographics.

	Placebo		LF	
Subjects, *n*	79		78	
Men, *n*	37		36	
Women, *n*	42		42	
Age (y), mean (SD ^1^) (range)	21.4 (3.2) (18–36)		21.6 (3.4) (18–38)	
Height (cm), mean (SD)	163.2 (8.3)		164.2 (9.0)	
Weight (kg), mean (SD)	56.5 (11.2)		58.5 (11.4)	
Frequency of experience of cold-like symptoms before and after COVID-19 pandemic	Before	After	Before	After
≥3/y, *n*	5	4	8	3
2/y, *n*	8	7	5	6
1/y, *n*	37	20	38	22
0/y, *n*	29	48	27	47

^1^ Standard deviation.

**Table 2:** Total scores of respiratory symptoms.

	Placebo	LF	*p* Value
Respiratory symptom score in 12 weeks, Median (IQR ^2^)	32.00 (15.00, 98.00)	11.00 (5.75, 27.50)	0.016
Week 1–4, Median (IQR)	14.00 (3.00, 25.25)	11.00 (3.50, 28.00)	0.705
Week 5–8, Median (IQR)	19.00 (10.50, 20.00)	4.00 (2.00, 14.00)	0.048
Week 9–12, Median (IQR)	42.00 (19.00, 68.75)	6.00 (4.00, 9.00)	0.002

^2^ Interquartile range (25th percentile–75th percentile).

**Table 3:** Total scores of systemic symptoms.

	Placebo	LF	*p* Value
Systemic symptom score in 12 weeks, Median (IQR)	30.00 (8.00, 55.50)	5.00 (3.00, 17.00)	0.045
Week 1–4, Median (IQR)	32.00 (5.00, 118.00)	4.00 (1.00, 25.00)	0.093
Week 5–8, Median (IQR)	6.00 (3.50, 11.00)	2.00 (1.25, 2.75)	0.010
Week 9–12, Median (IQR)	17.00 (4.00, 24.50)	4.00 (3.00, 8.00)	0.034

**Table 4:** CD86 and HLA-DR expression levels on pDCs.

		Placebo	LF	*p* Value ^5^	*p* Value ^6^	*p* Value ^7^
CD86 log_10_ MFI ^3^, Mean (SE ^4^)	Week 0	3.013 (0.016)	2.987 (0.017)			
	Week 12	2.960 (0.016)	2.963 (0.017)	0.032	<0.001	0.012
	∆12 weeks	−0.053 (0.008)	−0.024 (0.009)			
HLA-DR log_10_ MFI, Mean (SE)	Week 0	5.495 (0.013)	5.494 (0.014)			
	Week 12	5.401 (0.015)	5.441 (0.014)	0.014	<0.001	<0.001
	∆12 weeks	−0.093 (0.011)	−0.052 (0.013)			

^3^ Geometric mean fluorescence intensity; ^4^ standard error; ^5^ placebo vs. LF at week 12; ^6^ week 0 vs. week 12 in placebo; ^7^ week 0 vs. week 12 in LF.

## Data Availability

The data that support the findings of this study are available from the corresponding author upon reasonable request.

## Supplementary Materials

The following supporting information can be downloaded at: https://www.mdpi.com/article/10.3390/nu15183959/s1, Table S1: Cumulative number of days of respiratory and systemic symptoms. File S1: CONSORT 2010 checklist of information to include when reporting a randomised trial; CONSORT-Outcomes 2022 Extension Checklist Item.
