# Supplementary material for: Effects of Bovine Lactoferrin on the Maintenance of Respiratory and Systemic Physical Conditions in Healthy Adults—A Randomized, Double-Blind, Placebo-Controlled Trial
Source: Nutrients. 2023 Sep 13;15(18):3959. doi: 10.3390/nu15183959 (PMC10537451; doi:10.3390/nu15183959)
Supplement: Supplementary file 1 [file nutrients-15-03959-s001.zip › Table S1.pdf]

Table S1 Cumulative number of days of respiratory and systemic symptoms.

	**Placebo**	**LF**	**P value** [1]
Total	6468	5712	
Feeling feverish	68	38	0.024
Malaise	151	78	<0.001
Chill	69	24	<0.001
Hoarseness	76	34	<0.001
Cough	123	77	0.018
Runny nose	168	125	0.155
Plugged nose	117	55	<0.001
Head congestion	120	68	0.003

[1] placebo vs LF by Fisher's exact test
